# Chapter 5: Network Biology Approach to Complex Diseases

**DOI:** 10.1371/journal.pcbi.1002820

**Published:** 2012-12-27

**Authors:** Dong-Yeon Cho, Yoo-Ah Kim, Teresa M. Przytycka

**Affiliations:** National Center for Biotechnology Information, NLM, NIH, Bethesda, Maryland, United States of America; Whitehead Institute, United States of America; University of Maryland, Baltimore County, United States of America

## Abstract

Complex diseases are caused by a combination of genetic and environmental factors. Uncovering the molecular pathways through which genetic factors affect a phenotype is always difficult, but in the case of complex diseases this is further complicated since genetic factors in affected individuals might be different. In recent years, systems biology approaches and, more specifically, network based approaches emerged as powerful tools for studying complex diseases. These approaches are often built on the knowledge of physical or functional interactions between molecules which are usually represented as an interaction network. An interaction network not only reports the binary relationships between individual nodes but also encodes hidden higher level organization of cellular communication. Computational biologists were challenged with the task of uncovering this organization and utilizing it for the understanding of disease complexity, which prompted rich and diverse algorithmic approaches to be proposed. We start this chapter with a description of the general characteristics of complex diseases followed by a brief introduction to physical and functional networks. Next we will show how these networks are used to leverage genotype, gene expression, and other types of data to identify dysregulated pathways, infer the relationships between genotype and phenotype, and explain disease heterogeneity. We group the methods by common underlying principles and first provide a high level description of the principles followed by more specific examples. We hope that this chapter will give readers an appreciation for the wealth of algorithmic techniques that have been developed for the purpose of studying complex diseases as well as insight into their strengths and limitations.

**What to Learn in this Chapter**

- Characteristics and challenges of complex diseases
- Physical and functional networks and the methods to construct them
- Different classes of algorithms that use networks to leverage genotype, gene expression, and other types of data to identify dys-regulated pathways in diseases:
  - Scoring, correlation, and set cover based methods for identification of dys-regulated network modules based on various data types including genotype and phenotype.
  - Distance and flow based methods for inferring information flow from genotype to phenotype
- Applications to disease classification and treatment

This article is part of the “Translational Bioinformatics” collection for *PLOS Computational Biology*.

## 1. Introduction

*Complex diseases* are caused, among other factors, by a combination of genetic perturbations. Thus in the case of a complex disease we do not assume that a single genetic mutation can be pinned down as a cause. Many diseases fall in this category including cancer, autism, diabetes, obesity, and coronary artery disease. Even though there are other factors involved in such diseases, this review will focus on genetic causes.

One of the fundamental difficulties in studying genetic causes of complex diseases is that different disease cases might be caused by different genetic perturbations. In addition, if a disease is caused by a combinatorial effect of many mutations, the individual effects of each mutation might be small and thus hard to discover. For example, autism is considered to be one of the most heritable complex disorders, but its underlying genetic causes are still largely unknown [1]. One of the proposed factors that contribute to this difficulty is the role of rare genetic variations in the emergence of the disease [2].

An additional difficulty in studying complex diseases relates to disease heterogeneity. Specifically, in a complex disease, disease phenotypes might vary significantly among patients. The recognition of this fact has lead, for example, to renaming “autism” to “autism spectrum disorders” (ASDs) referring in this way to a group of conditions characterized by impairments in reciprocal social interaction and communication, and the presence of restricted and repetitive behaviors [1]. Similar heterogeneity is present in other complex diseases including cancer.

Given the above challenges, how can we approach the study of complex diseases? A useful clue is provided by the fact that genes, gene products, and small molecules interact with each other to form a complex interaction network. Thus a perturbation in one gene can be propagated through the interactions, and affect other genes in the network. However, the fact that we observe similar disease phenotypes despite different genetic causes suggests that these different causes are not unrelated but rather dys-regulate the same component of the cellular system [3]. Therefore in studies of complex diseases researchers increasingly focus on groups of related/interconnected genes, referred to as modules or subnetworks.

## 2. Interactome

Biomoecules in a living organisim rarely act individually. Instead, they work together in a cooperative way to provide specific functions. A variety of intermolecular interactions including protein-protein interactions, protein-DNA interactions, and RNA interactions are essential to these cooperative activities. These interactions can be conveniently represented as networks (graphs) with nodes (vertices) which denote molecules, and links (edges) which denote interactions between them. Depending on the type of interaction, the corresponding edge might be directed or undirected. For example, a binding between two proteins is usually represented as an undirected edge while an interaction between a transcription factor and a gene whose expression is regulated by the given transcription factor is usually represented as a directed edge where the direction goes from the transcription factor to the gene.

Biological interaction networks have characteristic topological properties [4]. One of the basic properties observed in many biological networks is the scale-free property [5]. A scale free network is defined as a network whose node degree distribution follows a power law. Formally, the function *P*(*k*) indicating the fraction of nodes interacting with *k* other nodes in the network follows *P*(*k*)∼α*k* ^−γ^, where α is a normalization constant and the degree exponent γ is usually in the range of 2<γ<3. Obviously, in biological networks the scale free property holds only approximately and practically the most important implication of this observation is the fact that these networks are characterized by a small number of highly connected nodes while most nodes interact with only a few neighbors. These highly connected nodes, called hubs have been proposed to play important roles in biological processes [6] and shown to be related to the modular structure of the physical and functional interaction networks [7]. Therefore it might be interesting to consider disease related genes in the context of the topological properties of interaction networks such as connectivity or modularity [8], [9]. With respect to connectivity, one should note that known disease genes tend to be more studied which might introduce a bias towards higher connectivity. Importantly, independently of the source of the non-uniformity of node degree distribution, this characteristic property of interaction networks needs to be kept in mind while designing proper null models for conclusions derived using these networks.

In the following subsections, we briefly describe how physical and functional interactions networks are constructed and how they are applied to analyze complex diseases. We then explore the modularity of networks – a widely accepted phenomenon in biological networks that has proven to be helpful in disease studies.

### 2.1 Physical Interaction Networks

Physical contacts between proteins are critical in many biological functions. In fact much of the molecular machinery responsible for transcription, translation, and degradation is made of stable protein complexes. There are two main approaches for detecting physical protein interactions [10]. The first approach is to detect physical interactions between protein pairs. The most widely used high-throughput technology for detecting pairwise interaction is yeast two-hybrid (Y2H) method. Alternatively, physical interactions among groups of proteins can be detected without explicit consideration of interacting partners. For this type of approach, interaction data is typically obtained by tandem affinity purification coupled to mass spectrometry (TAP-MS). A more detailed review on experimental methods for the detection and analysis of protein-protein interactions can be found in [11]. It is worth noting that networks obtained with various technologies often have different topological properties [7]. For example, in the case of the yeast TAP-MS network, hub nodes are enriched with essential genes (the genes without which yeast cannot survive in standard growth medium). In contrast, hubs in yeast Y2H networks are enriched with genes that are pleiotropic [12]. Finally, experimental procedures detecting protein-protein interactions have also been complemented by various computational methods using evolutionary-based approaches, statistical analysis, and/or machine learning techniques (for a review, see [13]).

While these physical interaction networks have significantly advanced our understanding of the relationships between molecules, a concern is their level of noise and incompleteness. Indeed, physical interaction networks obtained by high-throughput techniques are found to include numerous non-functional protein-protein interactions [14] and at the same time many missing true interactions. Therefore physical interactions are often complemented with functional interactions.

### 2.2 Functional Interaction Networks

While physical interaction networks provide information on how proteins interact with each other, sometimes we may be more interested in how proteins work together to perform a certain function. Functional networks aim to connect genes with similar or related functions even if they do not necessarily physically interact. Similarly functional regulatory networks are constructed so that the interactions depict direct or indirect regulatory relationships. Consequently, several computational methods have been proposed to derive functional interaction networks.

Since functionally related genes are likely to show mutual dependence in their expression patterns [15], gene expression data has been often used to detect functional relationships. Co-expression networks can be constructed by computing correlation coefficients or mutual information between gene expression profiles of every pair of genes in different experimental settings. To build more comprehensive functional networks, co-expression data is frequently combined with other types of data such as Gene Ontology [16], [17], outcome of genetic interaction experiments, and physical interactions. Such integrated networks have been constructed for a variety of organisms including yeast [18], fly [19], mouse [20], and human [21].

Gene regulatory network reconstruction algorithms such as ARACNE [22] and SPACE [23] identify regulatory relationships building on the assumption that changes in the expression level of a transcription factor should be mirrored in the expression changes of the genes regulated by the transcription factor (TF). Causal relations among genes can also be naturally modeled using Bayesian networks which can represent conditional dependencies between expression levels (for a primer on Bayesian network analysis utilizing expression data (see [24]); for a recent review see [25]). Considering the temporal aspects of gene expression profiles, dynamic Bayesian networks have been used to model feedback loops as well as gene regulation patterns [26], [27]. While expression profiles serve as primary data sources for constructing functional regulatory networks, this data is often complemented with additional information such as experimentally derived transcription factor binding data from ChiP-seq experiments or computationally identified binding motifs.

### 2.3 Modules and Pathways

It is widely accepted that the cellular system is modular. Hartwell *et al.* defined a functional module as an entity, composed of many types of interacting molecules, whose function is separable from those of other modules [28]. While the precise meaning of separation is left undefined, this general description provides a good intuition behind the concept of a module. Traditionally, molecular pathways have been delineated by focused studies of particular functions such as cell growth. Typically, these pathways contain not only topological connectivity information but also the roles of molecules such as whether a given molecule is an activator or inhibitor of the activity of another molecule. However, these hand-curated pathways are often incomplete. In addition, while some functions, such as cell growth or differentaition, have been relatively well studied, studies of other pathways are less extensive. Therefore, given the availability of large scale interaction networks, it is natural to attempt to extract meaningful functional modules from such networks. While there is no unique way to mathematically define functional modules, the most common approach is to search for densely connected subgraphs or clusters [29]–[46]. Additionally, gene expression information can be used alone or in concert with protein interaction data to obtain gene modules by grouping co-expressed genes into one module [47]–[49].

It is important to keep in mind that modules identified by analysis of high-throughput data are noisy, containing both false negative and false positive edges. In addition they do not usually provide information about the nature of an interaction. Therefore, unlike hand curated pathways, computationally identified network modules typically lack a mechanistic explanation of pathway activities but rather serve as groups of genes that work together to achieve a particular function.

An important advantage of working with modules rather than individual genes relates to the fact that it is often easier to predict the function of a module than the function of a gene. In particular, while the functions of many genes are still unknown, the prediction of the functional role of a module may be possible if the module contains a sufficient number of genes of known functions. Such enrichment analysis builds on the assumption that a fraction of genes can be assigned a functional category such as Gene Otology (GO) term [17]. The question of whether the number of genes with a functional annotation in a given gene module is higher than expected by chance can be determined by statistical tests such as *χ* ^2^ or Fisher exact test. A variety of software tools have been developed to perform such an analysis [50].

## 3. Identifying Modules and Pathways Dys-regulated in Diseases

Since complex diseases are believed to be caused by combinations of genetic alterations affecting a common component of the cellular system, module-centirc approaches are particulalry pormissing in thier study. How can disease associated modules/subnetworks be identified? Complementing interaction data with additional data related to disease states helps in separating subnetworks perturbed in a disease of interest from the remainder of the network. Both genotypic data (e.g., SNP, copy number alteration) and molecular phenotypic data such as gene expression profiles in disease samples have been used to aid the identification of perturbed network modules and explain the connection between genotypic and phenotypic data (reviewed in [51]). Basing on the assumption that complex diseases are caused by a set of mutations which, although strongly vary among patients, are likely to dys-regulate common pathways, such dys-regulated pathways might be uncovered by mapping genes altered in the diseases onto a PPI (protein-protein interaction) network and then searching for network modules enriched with the altered genes. On the other hand, organismal level phenotypes such as diseases are directly related to molecular level changes such as gene expression. Thus an alternative group of approaches considers modules enriched with abnormally expressed genes. Finally, molecular pathways can also be considered as means of information flow. For example, the activation of the EGFR signaling pathway starts with the activation of the EGFR receptor, which in turn activates a number of signaling proteins downstream which initiate several signal transduction cascades, such the MAPK, Akt and JNK pathways and culminate in cell proliferation. Thus the third type of approaches focuses on predicting molecules and modules that mediate such information propagation.

What are the benefits of analyzing phenotypic and genotypic differences in diseases in the context of their molecular interactions? First, the integrative network based approaches can identify subnetworks that include genes that do not necessarily show a significantly different state in disease versus control but still play an important role within a module by mediating a connection between other disease associated genes. For example, in their pioneering approach, Ideker *et al.* [52] integrated yeast protein–protein and protein-DNA interactions with gene expression changes in response to perturbations of the yeast galactose utilization pathway and identified *Active Subnetworks* (sets of connected genes with significantly differential expression) which included common transcription factors showing moderate changes in their gene expression level but connecting other dys-regulated genes. Second, a module based approach increases statistical power, allowing the identification of a perturbed module even in the case when the perturbation of each individual gene in the module might not be statistically significant. For example, many cases of genetic diseases such as autism and schizophrenia are affected by rare germline variations which are difficult to distinguish from noise due to their rarity. However, recent studies showed that a significant portion of the altered genes belong to a highly interconnected protein network [53], suggesting the network approach can better detect the causal genes. Third, identified network modules can provide better understanding of the biological underpinning of the diseases and therefore more reliable markers in disease diagnosis and treatments (see Section 4 for more discussion).

### 3.1 Network Modules Enriched with Genetic Alterations

**Figure 1:**
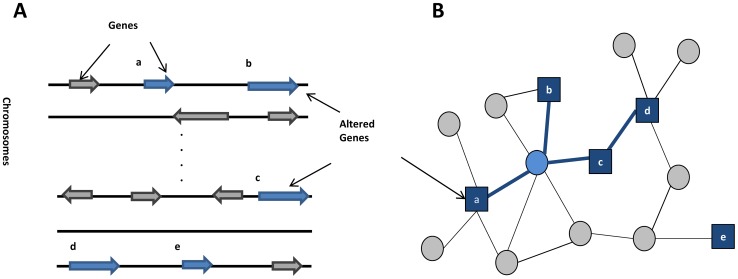
Identification of network modules enriched with genetic alterations. (A) Genomic regions with alterations. (B) Genes in the altered regions are mapped to the interaction network and modules enriched with such genes are identified.

One way in which differing genetic variations might dys-regulate a common pathway is when the genes containing these alterations belong to the pathway. This potential explanation has led to the idea that the dys-regulated pathways might be uncovered by mapping the genes altered in the diseases to an interaction network and searching for the modules enriched with the altered genes (See Figure 1).

Following this principle, the first step to identify such modules is to select candidate genes whose alterations may have caused a disease of interest. Genes or whole genomic regions that are altered in the disease are first identified, and the genes residing in the altered regions are mapped to an interaction network. Both physical and functional interaction networks can be used, and edges might be weighted based, for example, on the likelihood of having the same phenotypes or influences between genes [54]–[56]. Next, modules are typically defined as subsets of genetically altered genes that are highly interconnected or within close proximity to each other in the interaction network together with non-altered genes necessary to mediate these connections. Edge weights, if given, can be used to prioritize the modules. In many cases, finding the best subnetwork is computationally expensive and search algorithms such as greedy growth heuristics or more sophisticated approximation algorithms have been proposed. Finally, rigorous statistical tests have been applied to evaluate the significance of selected modules.

*Examples*. The idea of finding genetically altered network modules has been utilized in various disease studies. Analyzing ovarian cancer TCGA data (The Cancer Genome Atlas), HOTNET identified subnetworks in a protein interaction network in which genes are mutated in a significant number of patients [54]. The identified networks includes the NOTCH signaling pathway which is indeed known to be significantly mutated in cancer samples [57]. The method is based on the set cover approach (see Set cover based approach section below), which is found to be effective in capturing different genetic variations across patients. In the NETBAG (NETwork-Based Analysis of Genetic associations) method, developed by Gilman *et al.* and applied to identify a biological subnetwork affected by rare de novo copy number variations (CNVs) in autism [58], [59], the authors first constructed a gene network where edges were assigned the likelihood odd ratio for contributing to the same genetic phenotype. Subsequently a greedy growth algorithm was used to find clusters in this network. In another approach, Rossin *et al.* [60] considered the genomic regions found to be associated with Rheumatoid Arthritis (RA) and Crohn's disease (CD) in previous GWAS studies, and connected the genes residing in these regions based on interaction data to obtain network modules. It was also verified that those identified modules exhibited significant differences in expression level in the disease samples.

### 3.2 Differentially Expressed Network Modules

**Figure 2:**
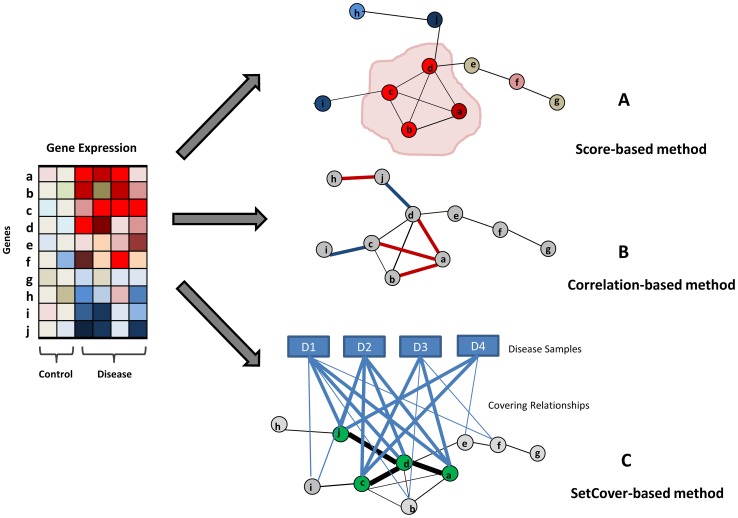
Finding differentially expressed modules. (A) Score based method selects the module with significant expression changes. (B) Correlation based method selects edges with correlation changes. The red and blue edges are correlated and anti-correlated edges, respectively. (C) Set cover based method selects a set of genes covering all samples. In this example, each sample has at least 2 differentially expressed genes and the genes are connected in the network.

Another popular and successful approach to find disease associated modules is to search for subnetworks that are significantly enriched with genes whose expression levels are changed in disease samples. Building on the observation that a molecular perturbation typically affects the expression levels of genes in a whole module rather than individual genes, these approaches identify the modules which exhibit different expression patterns in disease states relative to a control. Gene expression data has been widely utilized for identifying dys-regulated modules and drug targets, inferring interactions between genes, and classifying diseases. While these approaches are based on the common idea of finding gene modules enriched with genes that have abnormal expression, several different computational techniques have been used to achieve these tasks, which we discuss shortly below. The methods are also illustrated in Figure 2.

#### 3.2.1 Scoring based methods

Suppose that there is a subset of genes which are differentially expressed in disease samples and they are closely connected to each other in an interaction network. A subnetwork including such genes might be a good candidate for a disease associated network module (Figure 2A). Implementing this idea requires a way to score candidate modules. Various methods have been suggested for measuring the significance of the differential expression of genes in a module and their connectivity (the distances between the genes). In addition, different methods adopt different search algorithms to find high scoring candidate modules. Finally, some approaches additionally require that all genes are either up-regulated or down-regulated in the same direction.

*Examples*. Chuang *et al.* defined the activity score for a subnetwork by comparing gene expression profiles from two different types of samples (metastatic or non-metastatic in their study) [61]. More specifically, they first computed how well the expression of a gene discriminates between the two patient groups and then scored candidate subnetworks based on aggregate discriminative power over all genes in the subnetwork. Then they searched for the most discriminative networks in a greedy manner. While the method was used for disease classification (see Section 4), it can readily be applied to leverage the difference between disease and non-disease cohorts.

#### 3.2.2 Correlation based methods

Comparing expression patterns between genes is a basis for constructing a co-expression network, extracting modules exhibiting similar expression patterns, and further understanding molecular changes in diseases. Considering expression correlation of disease cases in the context of interactions can provide additional power in the identification of a disease associated module (Figure 2B). If the expression changes of two neighboring nodes are correlated with each other, this may suggest that the two interacting genes have related functional roles. With this in mind, some approaches look at connected components which show highly correlated and anti-correlated expression patterns. Other approaches search for loss and gain of correlation in disease states to identify dys-regulated edges.

*Examples*. Aiming to identify regulatory networks defining phenotypic classes of human cell lines, Müller *et al.* searched for Jointly Active Connected Subnetworks (connected subnetworks with high average internal expression similarity) in a human interaction network [62] and demonstrated the power of combining network and expression data.

IDEA (Interactome Dysregulation Enrichment Analysis) method [63] focused on the identification of perturbed network edges in a combined interaction network (PPI, transitional, signaling, posttranslational modifications predicted by MINDy [64]), and searched for the edges connecting genes which in a disease state show loss or gain of expression correlation. The utility of the method was demonstrated in the analysis of FL lymphoma and other cancer types. In particular, they identified BCL2 as the gene adjacent to the largest number of dys-regulated edges in FL lymphoma. This analysis also identified the SMAD1 gene, which could not be detected by differential expression analysis only.

To understand the mechanism of aging, Xue *et al.* applied a network module approach [65], [66]. They utilized a PPI network and overlaid expression data obtained from various stages of aging. Two types of edges – correlated and anti-correlated – were selected. The subnetwork that includes only those edges was called the NP (negative and positive) network, is proposed to be related to the aging mechanism. Further modularizing the network with hierarchical clustering of expression patterns, they obtained a few age related modules and found some genes connecting different modules through PPIs are more likely to affect aging/longevity, which was also experimentally validated.

#### 3.2.3. Set cover based methods

A group of methods employ a combinatorial approach named set cover. In a set cover, a gene is considered to cover a disease sample if it is dys-regulated in the sample. For example, it can be decided if a gene is covering a sample or not based on the fold change of gene expression level in the sample or using a statistical test such as z-test. The main principle of the set cover approach is that each disease case has some dys-regulated (thus covering) genes but in heterogeneous diseases, different cases will typically have different covering genes. Set cover approaches provide a strategy to select a representative set of such covering genes (Figure 2C). This is usually done by defining some optimization criterion and attempting to select a set of genes which is optimal with respect to this criterion. For example, given a set of genes and disease samples along with covering relationships, a subset of genes is selected so that each sample is covered by some minimal number of genes while the total number of selected genes is minimized.

Many observed organism-level phenotypes arise in a heterogeneous way. Diseases such as cancer are now seen as a spectrum of related disorders that manifest themselves in a similar fashion. Since different samples may be covered by different genes and those genes may be connected in an interaction network, set cover approaches can be useful to identify gene modules explaining a heterogeneous set of samples [67]–[69].

*Examples*. Aiming to detect dys-regulated pathways in complex diseases, Ulitksy *et al.* extended the set cover technique by integrating expression data and interaction networks [67]. Their method, named DEGAS (de novo discovery of dys-regulated pathways) searches for a smallest set of genes forming a connected subnetwork so that each disease sample is covered by a certain minimal number of genes. They applied this approach to a Parkinson's disease dataset. Chowdhury *et al.* [68], developed an alternative network cover based algorithm and used the identified modules for disease classification in a human colorectal cancer dataset.

Set Cover approaches have also been applied to data types other than gene expression. For example, Kim *et al.* proposed a module cover approach to identify gene modules which collectively cover disease samples [70]. At the same time they required that each module is coherent, containing genes with similar genotype-phenotype mappings (see Section 4 for more discussion). The HotNet Algorithm discussed in Section 3.1 also utilized a variant of a set cover approach to find genetically altered modules. In their case, a gene is defined to cover a sample if the gene is mutated in the sample, and they looked for a fixed size connected set of genes covering as many samples as possible. The Dendrix (De novo Driver Exclusivity) algorithm was also developed to discover mutated gene modules in cancer and, though it does not utilize interaction data, it aims to find sets of genes, domains, or nucleotides whose mutations exhibit both high coverage and high exclusivity in the disease samples [71].

### 3.3 Uncovering Information Propagation Modules

**Figure 3:**
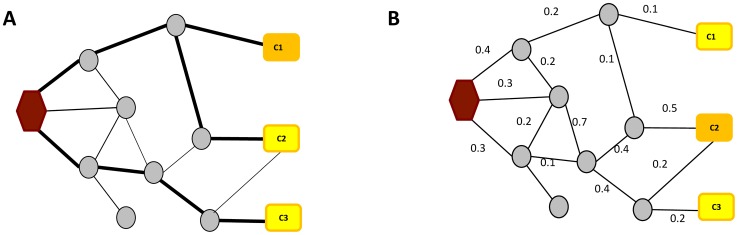
Finding information propagation modules. (A) Shortest path approach to uncover information propagation. The shortest paths from a target gene (with hexagon shape) to each of three candidate genes are shown. The closest gene is identified as the most probable disease causing gene. (B) Flow based approach. The gene receiving the most significant amount of flow is identified as the disease gene. The information flow methods often follow Kirchhoff's current law (the amount of incoming information equals the amount of outgoing information).

The approaches discussed thus far have dealt with modules of genes associated with either phenotypic or genotypic information. While both approaches are helpful for predicting dys-regulated modules, a more effective way to understand disease mechanisms might be to combine both genotypic (the putative causes of diseases) and phenotypic data (their effects). Expression Quantitative trait loci (eQTL) analysis is a useful method to find the relationship between genotype and phenotype [72], [73]. eQTL treats the level of gene expression as a quantitative phenotype, which is assumed to be controlled by genotypic information. Loci that putatively control the expression of a given gene are identified by determining the associations between genotype and gene expression. Given an association between a genotypic variation in a locus and expression level of a gene, the next challenge is to uncover the pathway(s) through which the genetic variation leads to the expression change. Recently, several groundbreaking pathway elucidation methods have emerged, as illustrated in Figure 3 and described below.

#### 3.3.1 Distance based methods

A simple approach to identify a possible pathway from a genetically altered gene (putative cause) to the gene with correlated expression change (target gene) is to test if there is a path in an interaction network connecting the putative causal gene to its target gene. The shortest path connecting a causal gene and its target is often used to explain their causal relationship (Figure 3A). The intermediate nodes on such a shortest path are likely members of an affected pathway/module. Several variations of the shortest path approach have been used in extracting disease associated network modules [74]–[76]. For example, Carter *et al.* searched for the shortest directionally consistent paths in molecular interaction networks connecting seed genes to their targets. The targets were inferred by linear decomposition of gene expression data [76].

When multiple target genes exist, the well-known graph-theoretical concept of a Steiner tree is often used in place of a set of shortest paths. Given a set of nodes to be connected, a Steiner tree is an acyclic subgraph (a tree) connecting all these nodes while using the minimum number of edges. In a Steiner tree, the individual path from the putative causal gene (the root of the tree) to each of the target genes does not need to be the shortest, but the size (i.e., the number of edges) of the whole tree is minimized. The Steiner tree approach has been used to find new functional associations for proteins [77]. Tuncbag *et al.* extended the approach to the Steiner forest problem (allowing multiple trees), applying it to proteomic data from glioblastoma multiforme (GBM). In their study, each tree was rooted in a different cell surface receptor and represented independent signaling pathways originated from this receptor [78].

Distance based methods, such as the shortest path approach or the Steiner tree method, have several shortcomings. In particular, they ignore the fact that a pair of genes may have multiple paths connecting them in a network. In addition they use network topology without considering additional data (e.g. gene expression) and assume that the shortest pathways are the most informative or most likely used paths, which may not always be the case.

#### 3.3.2 Flow-based methods

In the information flow approach, genotypic variations are considered the source of perturbation, while genes with phenotypic changes are considered the targets of a perturbation pathway. Instead of finding single paths connecting source and targets, flow-based methods compute the fraction of flow going through each intermediate node/edge. Fraction of flow indicates the probability of using the given path in information propagation (Figure 3B). In the case of current flow approach, the network is modeled to mimic the behavior of current in an electronic circuit, where each edge has an associated resistance. The current flow network provides an efficient framework equivalent to a random walk, which is also often used for modeling information flow in biological networks (see discussion below). An important advantage of network flow approaches their ability to incorporate additional data (such as gene expression, confidence level of interactions, and functional associations of genes) to the probabilistic network models. By incorporating such additional data, network flow approaches can more confidently suggest information propagation pathways.

The information flow of biological networks has been used to predict protein functions, to prioritize candidate disease genes, and to find network centralities [7], [79]–[88]. The flow-based approach is particularly useful for augmenting network information for eQTL analysis. Specifically, it can be used to pinpoint likely causal genes in genomic eQTL regions and to uncover genes involved in the propagation of information signals from such causal genes to their target genes.

There are several mathematical formulations that can be used to capture information propagation. In addition to the aforementioned current flow, other approaches include random walk and network flow. While mathematically different, many information propagation methods share a number of similar assumptions such as flow conservation (Kirchhoff's law). In the random walk method, a number of random walkers repeatedly start from a node. The likelihood of associating a gene in the network to a disease is estimated by the number of random walkers arriving at the gene. Gene expression correlation provides one way to compute the weight of a gene in the network which, in turn, provides the transition probability of the random walker. The network flow methods are closely related to the current flow approach. Unlike current flow, however, the network flow model resembles water-finding paths through pipes. Capacities are associated with pipes (edges) providing constraints on how much flow can go through each pipe.

*Examples*. Tu *et al.* [79] used the random walk approach to infer causal genes and underlying causal paths over a molecular interaction network for yeast knock-out experimental data. Current flow is an equivalent form of random walk that can be used in a more computationally efficient way [89]. Using this knowledge, Suthram *et al.* [80] developed the eQED method, which integrates eQTL analysis with molecular interaction information modeled as a current flow network.

Kim *et al.* further extended the eQED idea to identify causal genes and dys-regulated pathways and applied it to Glioma sample analysis [69], [90].One of the challenges of eQTL analysis is a massive multiple testing problem, for which various multiple testing correction methods have been proposed. Without such corrections, eQTL analysis typically finds multiple associated regions for each target gene, many of which are simply by chance. However, simply applying a more stringent p-value cutoff for multiple testing corrections often eliminates many true causal regions. Moreover, each region may contain dozens of candidate causal genes. Current flow analysis can be applied to complement eQTL analysis and help to identify the genes whose alterations are most likely to cause abnormal expression for the target gene. Using copy number variations and gene expression profiles of the same set of cancer patients, Kim *et al.* first identified chromosomal regions where copy number variations correlated with gene expression changes. Subsequently, they used the current flow algorithm to identify potential causal genes in the associated regions. By selecting genes receiving significant amounts of current in the network, Kim *et al.* identified putative causal genes in Glioblastoma and uncovered commonly dys-regulated pathways, including insulin receptor signaling pathways and RAS signaling. The identified pathways featured several hub nodes, such as EGFR, that were known to be important players in Glioma or more generally in cancer. Compared to simple genome-wide association studies, which only identify putative associations between causal loci and target genes, the current flow based method provides increased power to predict causal disease genes and to uncover dys-regulated pathways.

A variant of the network flow approach, the minimum cost network flow, was used to model the response to increased expression of alpha-synucleain, a protein implicated in several neurodegenerative disorders, including Parkinson's disease [81]. In addition to the edge capacities, the min cost network flow approach associates weights with edges representing the cost of sending flow through an edge. These weights were computed based on the probability of the two genes interacting in a response pathway, while capacities were calculated using the transcript levels of target genes.

## 4. Applications of Network Modules – Disease Diagnosis and Treatment

Can network modules help facilitate a more personalized approach for disease diagnosis and treatment? Traditional approaches of clinical disease classification have been based on pathological analysis of patients and existing knowledge of diseases. However, traditional diagnostic approaches are prone to errors. Alternatively, knowledge about dys-regulated pathways can be used to subtype diseases and to develop relevant treatments for individual disease subgroups. For example, network modules have been used to predict patient survival, metastasis, drug responses for various types of cancer [61], [68], [91]–[94].

### 4.1 Disease Classification

A supervised approach to disease classification starts with a set of samples with a known partition into disease subtypes (e.g., metastatic or not) and attempts to identify a classifying principle using specific molecular features. The general strategy for supervised disease classification is to search for subnetworks, also called subnetwork markers, whose activities best discriminate the two disease subtypes. As in the case of single-gene disease markers, a network marker will distinguish some but not all disease cases and multiple subnetworks might be necessary. Among selected candidate network markers, the best markers are selected based on a set of training samples. Some methods take an unsupervised approach, where subclasses and their features are discovered without using a known training set.

*Examples*. Chuang *et al.* showed how dys-regulated network modules (described in Section 3.2) provide more robust and accurate predictions than those by single gene based classifications when applied to breast cancer metastasis analysis [61]. Chuang *et al.*'s work provided the proof of principle for using network modules in disease classification. A number of subsequent extensions and improvements to Chuang *et al.*'s work were suggested. For example, Lee *et al.* incorporated curated pathways, and searched for a subset of genes with discriminative features for the disease phenotype [94]. More recently, Dao *et al.* developed alternative network based approaches for classification of cancer subtypes by identifying densely connected subnetwork and randomized algorithms [92], [93]. Other techniques for best marker identification, such as set cover and bottom-up enumeration techniques, were also proposed [68], [91].

Kim *et al.* identified gene modules using a module cover approach to capture disease heterogeneity in brain cancer samples from Rembrandt and Ovarian Cancer samples from TCGA [70]. Next, Kim *et al.* superimposed the selected modules onto the results from an independently proposed classification scheme [57]. As a result, Kim *et al.* uncovered which disease classes are characterized by which combinations of modules.

### 4.2 Disease Similarity

Network modules can also be used to explain disease similarity. Overlaps of dys-regulated network modules explain why some complex diseases share similar phenotypic traits. Suthram *et al.* used a variant of PathBlast [95] to identify dense subnetworks. Analysis of disease similarity was achieved by comparing expression patterns of various diseases in the modules [96]. Several dys-regulated modules were found to be common to many diseases, which explains why some drugs can treat many different diseases.

### 4.3 Response to Treatment

Modules may help determine whether a given patient will respond to a particular drug, which is valuable for treatment design. In addition, understanding molecular differences between responders and non-responders is likely to help development of alternative treatments. For example, Chu and Chen used a network approach to discover apoptosis drug targets [97]. Chu and Chen constructed a PPI network for apoptosis in normal cells and applied a nonlinear stochastic model to remove false positive interactions using microarray data. Comparing the resulting subnetworks helped to shed some light on the mechanisms leading to apoptosis and to identify potential drug targets.

## 5. Summary

Network biology provides powerful tools for the study of complex diseases. Network-based approaches leverage the idea that complex diseases can be better understood from the perspective of dys-regulated modules than at the individual gene level. Modularity is a widely accepted concept in molecular networks and module-based approaches provide a number of advantages including robustness in the identification of dys-regulated pathways and improved disease classification.

In addition, network based formulations allow using a wealth of methods already developed in graph theory, such as shortest paths, network flow, and Steiner trees. Network-based methods have several limitations including the lack of mechanistic explanations. Despite the limitations, network analysis has been applied successfully in many disease studies, suggesting testable hypotheses.

## 6. Exercises

**Figure 4:**
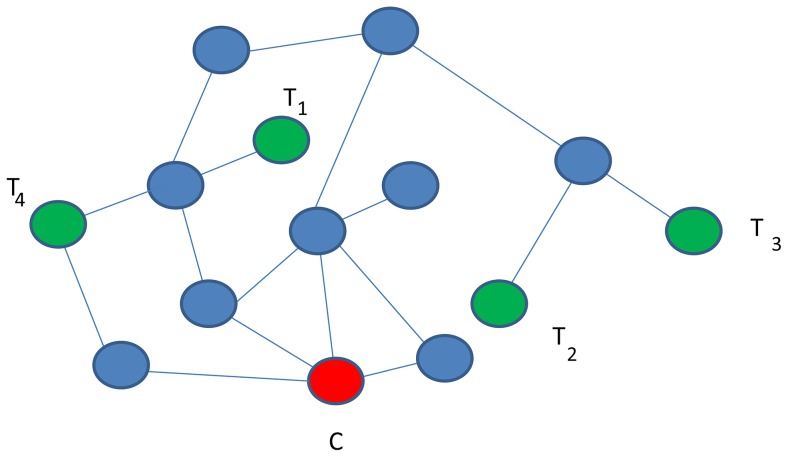
A hypothetical interaction network to be used with Exercises 5 and 6.

1. Construct coexpression networks following the steps below [98].
  - Download the three expression datasets from the following page: http://www.geneticsofgeneexpression.org/network/download
  - Compute 3 population-specific correlations for each pair of 4238 genes with the expression data. (Hint: There are 8,978,203 pairs of genes.)
  - For gene pairs which have similar correlations in the 3 datasets, calculate the weighted average correlation, weighted by the number of individuals in each population. Hint: In the Supplemental Table 1 published with [98] (http://genome.cshlp.org/content/suppl/2009/10/02/gr.097600.109.DC1/nayak_supplemental_material.pdf), you can find the list of gene pairs whose correlations differ significantly among the 3 datasets.
  - Construct the correlation network by connecting gene pairs whose weighted average correlations are greater than a pre-defined threshold (e.g., 0.5).
  - Compute specific parameters describing the network topology. (Hint: You can use the NetworkAnalyzer Cytoscape plugin http://med.bioinf.mpi-inf.mpg.de/netanalyzer/)
  - For different correlation thresholds, compare the networks in terms of topological properties.
2. Suppose that in a co-expression network two genes are identified to have correlated expression patterns. Provide at least two possible biological explanations of this correlation.
3. Some variants of information flow approaches that identify pathways of information flow from a mutated gene to a target gene with correlated expression require that the last but one node gene on such a pathway (the node preceding the target gene) to be a transcription factor. What is a justification for such requirement? What can be advantages and disadvantages of such a design?
4. Consider a set cover approach to find a representative set of genes dys-regulated in a given set of cancer patients. The algorithm finds the smallest number of genes so that each disease case is covered at least k times. How does the number of selected genes depend on k? If you suspect that data for 5% patients might be incorrect, how would you modify the optimization problem?
5. A Steiner tree connecting a set of nodes does not need to be unique. In the graph shown in Figure 4, find two different Steiner trees connecting genes C, T_1_, T_2_, T_3_, and T_4_.
6. In the graph shown in Figure 4, find the shortest paths connecting C with each of T_1_, T_2_, T_3_, and T_4_. Do the edges used by these paths correspond to a Steiner tree? Explain why or why not.

Answers to the exercises are provided in Text S1.

**Further Reading**

- Schadt EE (2009) Molecular networks as sensors and drivers of common human diseases. Nature 461(7261): 218–223.
- Barabási AL, Gulbahce N, Loscalzo J (2011) Network medicine: a network-based approach to human disease. Nat Rev Genet 12(1): 56–68.
- Przytycka TM, Singh M, Slonim DK (2010) Toward the dynamic interactome: it's about time. Brief Bioinform 11(1): 15–29.
- Przytycka TM, Cho DY (2012) Interactome. In: Meyers RA, editor. Encyclopedia of molecular cell biology and molecular medicine. John Wiley and Sons, Inc. doi:10.1002/3527600906.mcb.201100018
- Califano A, Butte AJ, Friend S, Ideker T, Schadt E (2012) Leveraging models of cell regulation and GWAS data in integrative network-based association studies. Nat Genet 44(8): 841–847. doi:10.1038/ng.2355
- Vidal M, Cusick ME, Barabási AL (2011) Interactome networks and human disease. Cell 144(6): 986–998.
- Kim Y, Przytycka TM (2012) Bridging the gap between genotype and phenotype via network approaches. Frontiers in Genetics special issue on mapping complex disease traits with global gene expression. Front Genet 3: 227. doi:10.3389/fgene.2012.00227

## Supporting Information

Text S1 Answers to Exercises.
(PDF)
