# Supplementary material for: Chapter 5: Network Biology Approach to Complex Diseases
Source: PLoS Comput Biol. 2012 Dec 27;8(12):e1002820. doi: 10.1371/journal.pcbi.1002820 (PMC3531284; doi:10.1371/journal.pcbi.1002820)
Supplement: Text S1 — Answers to Exercises. (PDF) [file pcbi.1002820.s001.pdf]

Network Biology Approach to Complex Diseases

Answers to exercises

Dong-Yeon Cho[1], Yoo-Ah Kim[1], Teresa M. Przytycka*

1. Construct coexpression networks [98]

   a. Download the three expression datasets from the following page:
      http://www.geneticsofgeneexpression.org/network/download
      There are 3 files which contain expression levels for 3 different populations.

   b. Compute 3 population-specific correlations for each pair of the 4238 genes with expression data.
      (Hint: There are 8,978,203 pairs.)
      For each population, the Pearson correlation coefficient between every pair of genes (x, y) can be calculated as follows:

$$r_{xy} = \frac{n\sum x_i y_i - \sum x_i \sum y_i}{\sqrt{n\sum x_i^2 - (\sum x_i)^2}\sqrt{n\sum y_i^2 - (\sum y_i)^2}},$$

      where n is the number of measurements for each population.

   c. For gene pairs which have been found to have similar correlations in the 3 datasets, calculate the weighted average correlation, weighted by the number of individuals in each population.
      (Hint: In the Supplemental Table 1, published with [98] (http://genome.cshlp.org/content/suppl/2009/10/02/gr.097600.109.DC1/nayak_supplemental_material.pdf), you can find the list of gene pairs whose correlations differ significantly among the 3 datasets.)
      For every pair of genes (x, y), the weighted average correlation can be calculated as follows:

$$r_{xy}^{WA} = \frac{87r_{xy}^{ASN} + 148r_{xy}^{CEPH-Utah} + 60r_{xy}^{YRI}}{(87+148+60)},$$

      where 87, 148, 60 are the numbers of samples in each population. However, the gene pairs in the Supplemental Table 1 have significantly different expression correlations among 3 datasets. Thus, these gene pairs should be excluded from the result so that we have the weighted average correlations for 8,968,248 pairs of genes.

[1] These authors contributed equally

* *Corresponding author e-mail przytyck@ncbi.nlm.nih.gov*

d. Construct the correlation network by connecting gene pairs whose weighted average correlations are greater than the pre-defined threshold (e.g., 0.5).

After selecting gene pairs whose weighted average correlations exceed the pre-defined threshold (e.g., $|r_{xy}^{WA}| > 0.5$), import the selected pairs into Cytoscape (File → Import → Network from Table) so that the coexpression network is constructed.

e. Compute specific parameters describing the network topology.

(Hint: We can use the NetworkAnalyzer Cytoscape plugin http://med.bioinf.mpi-inf.mpg.de/netanalyzer/)

Using NetworkAnalyzer plugin (Plugins → Network Analysis → Analyze Network), we can obtain the following parameters which describe the network topology:

- Clustering coefficient: 0.394
- Network density: 0.009
- Network diameter: 11
- Network heterogeneity: 1.224
- Network centrality: 0.054
- Characteristic path length: 3.602
- Average number of neighbors: 26.969

f. For the different correlation threshold, compare the networks in terms of topological properties.

Threshold	Clustering coefficient	Network density	Network diameter	Network heterogeneity	Network centrality	Characteristic path length	Average #neighbors
0.5	0.394	0.009	11	1.224	0.054	3.602	26.969
0.6	0.370	0.008	18	1.606	0.055	5.271	11.409
0.7	0.322	0.024	21	1.822	0.135	4.802	11.035
0.8	0.557	0.159	5	1.015	0.324	1.754	18.000
0.9	0.583	0.244	4	0.718	0.462	1.720	7.5625

2. Suppose that in a co-expression network two genes are identified to have correlated expression patterns. Provide at least two possible biological explanations of this correlation.

(i) One gene can be a transcription factor regulating another gene
(ii) Both genes are regulated by the same transcription factor.
(iii) Members of the same protein complex or pathway are often (directly or indirectly) co-regulated.

3. Some variants of information flow approaches that identify pathways of information flow from a mutated gene to a target gene with correlated expression require that the last but one node gene on such a pathway (the node preceding the target gene) to be a transcription factor. What is a justification for such requirement? What can be advantages and disadvantages of such a design?

It is reasonable to assume that the expression change of a gene is a direct consequence of the activity of a transcription factor. However we don't know transcription factors for most of the human genes which limits applicability of such approach to the human interactive.

4. Consider a set cover approach to find a representative set of genes dys-regulated in a given set of cancer patients. The algorithm finds the smallest number of genes so that each disease case is covered at least k times. How does the number of selected genes depend on k?

Increasing k will increase the number of genes required for the cover. In particular if there are k_1 genes that are dis-regualted in all patients, then for $k \leq k_1$ we will get k of such genes in the covering set. For $k > k_1$ different samples will have to be covered with different genes so the number of gene need to cover all cases k time will be larger than k.

If you suspect that data for 5% patients might be incorrect, how would you modify the optimization problem?

In such situation, it is reasonable to modify the set cover definition to require that all but 5% of cases are required to be covered k-times.

5. A Steiner connecting a set of nodes does not need to be unique. In Figure 4, Find two different Steiner trees connecting genes C,T_1,T_2, $T_{3,}$ T_4.

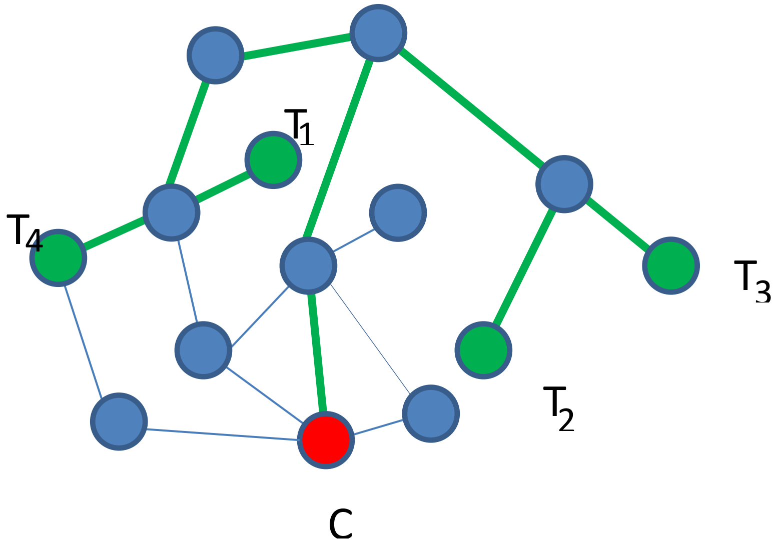

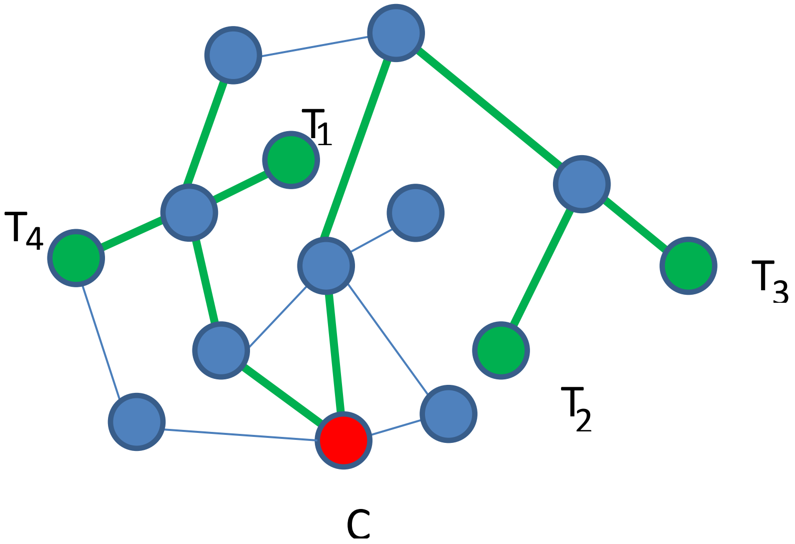

Two Steiner trees connecting genes C,T_1,T_2, $T_{3,}$ T_4 are shown in green. Both trees use 9 edges.

6. In the graph shown in Figure 4, find the shortest paths connecting C with T_1,T_2, T_3, T_4. Do the edges used by these paths correspond to a Steiner tree?

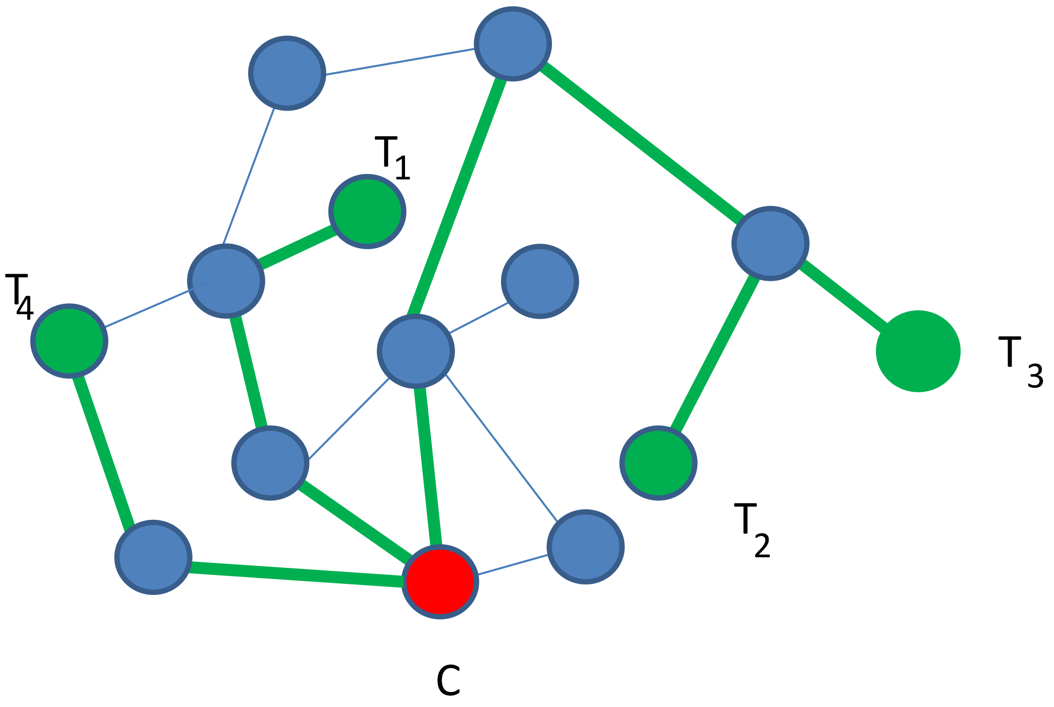

The shortest paths connecting C with T_1,T_2, T_3, and T_4 are shown in green. The edges used by these paths do not correspond to a Steiner since the do not minimize the number of edges needed to connect all nodes.
